# Maintenance of cell fates and regulation of the histone variant H3.3 by TLK kinase in *Caenorhabditis elegans*

**DOI:** 10.1242/bio.038448

**Published:** 2019-01-11

**Authors:** Yukimasa Shibata, Yoshiyuki Seki, Kiyoji Nishiwaki

**Affiliations:** School of Science and Technology, Department of Bioscience, Kwansei Gakuin University, 2-1 Gakuen, Sanda, Hyogo 669-1337, Japan

**Keywords:** Tousled-like kinase, CAF1, SIN3, BET, MYST HAT

## Abstract

Cell-fate maintenance is important to preserve the variety of cell types that are essential for the formation and function of tissues. We previously showed that the acetylated histone-binding protein BET-1 maintains cell fate by recruiting the histone variant H2A.z. Here, we report that *Caenorhabditis elegans* TLK-1 and the histone H3 chaperone CAF1 prevent the accumulation of histone variant H3.3. In addition, TLK-1 and CAF1 maintain cell fate by repressing ectopic expression of transcription factors that induce cell-fate specification. Genetic analyses suggested that TLK-1 and BET-1 act in parallel pathways. In *tlk-1* mutants, the loss of SIN-3, which promotes histone acetylation, suppressed a defect in cell-fate maintenance in a manner dependent on MYST family histone acetyltransferase MYS-2 and BET-1. *sin-3* mutation also suppressed abnormal H3.3 incorporation. Thus, we propose a hypothesis that the regulation and interaction of histone variants play crucial roles in cell-fate maintenance through the regulation of selector genes.

## Abstract (precis)

**Summary:** Histone H3 chaperone CAF1 maintains cell fate by repressing ectopic expression of genes for cell fate-specifying transcription factors. Accumulation of histone variant H3.3 correlates with defects in cell-fate maintenance.

## INTRODUCTION

Defects in cell-fate maintenance cause aberrant cell-fate transformation, which can induce tumor formation and tissue malfunction. Conversely, suppression of the mechanisms that maintain cell fate is necessary for efficient reprogramming such as the generation of induced pluripotent stem (iPS) cells (Takahashi and Yamanaka, 2015). Aberrant activation of genes that induce specific cell fates causes abnormal cell-fate transformation (Riddle et al., 2013; Halder et al., 1995; Tursun et al., 2011). Thus, the repression of the genes that specify cell fate is critical for maintaining individual cell fate.

Epigenetic marks including histone modifications play important roles in transcriptional repression during development. For example, methylation on lysine 27 of histone H3 (H3K27me) is required to silence developmentally regulated genes such as Hox genes (Ringrose and Paro, 2004). In contrast, the roles of histone variants in transcriptional repression are poorly understood. We previously showed that a histone H2A variant, H2A.z, is required to maintain cell fate in multiple cell lineages in *Caenorhabditis elegans* (Shibata et al., 2014). Subnuclear localization of H2A.z is regulated by an acetylated histone-binding protein, BET-1, which is also required to maintain cell fate. BET-1 represses selector genes that encode DNA-binding transcription factors (TFs) such as LIM homeodomain protein MEC-3 and CEH-22/Nkx2.5, which induce specific cell fates (Shibata et al., 2014, 2010; Shibata and Nishiwaki, 2014). The selector gene activates transcription of itself and of genes that are required for the specific function of each cell type (Hobert, 2008). Thus, although many studies suggest a role for H2A.z in transcriptional activation, H2A.z also preserves transcriptional repression in the maintenance of cell fate.

In addition to the H2A variant, another major histone variant is the histone H3 variant H3.3, which is often observed on actively transcribed loci (Wirbelauer et al., 2005). Canonical histone H3 and H3 variant H3.3 are deposited by chromatin assembly factor 1 (CAF1) and histone regulator A (HIRA), respectively (Tagami et al., 2004). In cultured cells, CAF1 depletion causes alternative deposition of H3.3 to fill the nucleosome gap at the replication site by HIRA (Ray-Gallet et al., 2011). CAF1 deficiency promotes artificial trans-differentiation, such as induction of iPS cells and the generation of neurons from fibroblasts and of macrophages from pre-B cells (Cheloufi et al., 2015). However, the roles of CAF1 and H3.3 in cell-fate maintenance during development are not known.

Tousled-like kinases (TLKs) are conserved protein kinases in multicellular organisms. They phosphorylate anti-silencing factor 1 (ASF1), which interacts with CAF1 (Klimovskaia et al., 2014). Arabidopsis TLK, Tousled, acts in the maintenance of transcriptional gene silencing and is required for leaf and flower development (Roe et al., 1993; Wang et al., 2007). In *C. elegans* early embryos, the ortholog TLK-1 is required for chromosome segregation and cytokinesis and promotes transcription (Yeh et al., 2010; Han et al., 2003, 2005). The *C. elegans* CAF1 complex is required to establish bilateral asymmetry (Nakano et al., 2011), although the *in vivo* relationship between TLK and the CAF1 complex is not known. In addition, the role of TLK and the CAF1 complex in cell fate maintenance remains elusive. Our genetic screening for mutants that are defective in cell-fate maintenance resulted in the isolation of *tlk-1* mutants. Here, we analyzed the roles of TLK-1 and CAF1 in cell-fate maintenance and the regulation of H3.3.

## RESULTS

### Isolation of *tlk-1* mutants by screening for cell-fate maintenance-defective mutants

**Fig. 1.**
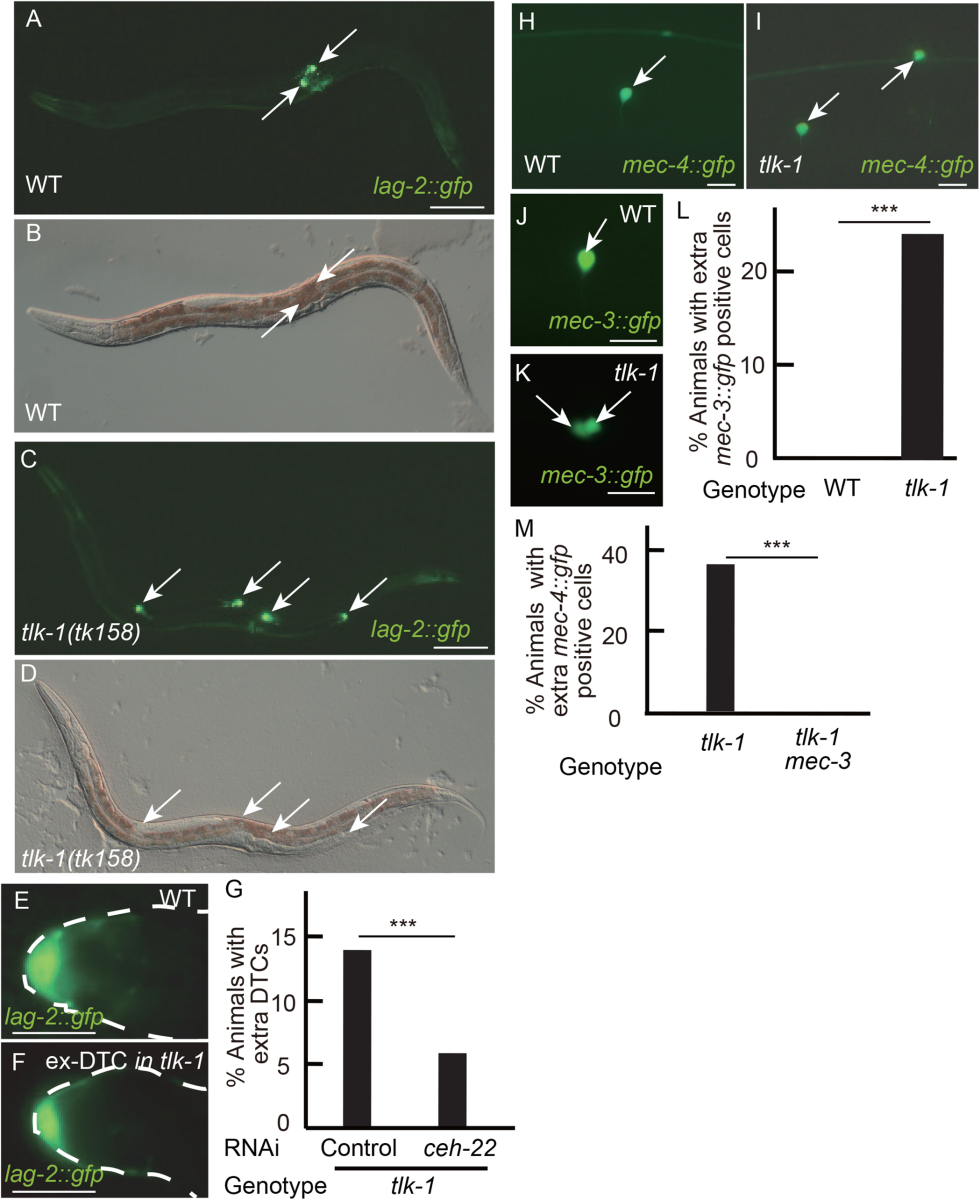
***tlk-1* functions in multiple cell types.** (A–F,H–K) GFP (A,C,E,F,H–K) and differential interference contrast (DIC) (B,D) images showing the expression of the DTC marker *lag-2::gfp* (A,C,E,F), *mec-4::gfp* (H,I), and *mec-3::gfp* (J,K) in wild type (WT) (A,B,E,H,J) and *tlk-1* mutants (C,D,F,I,K) at the adult stage. Anterior is to the left, ventral is to the bottom (A–D). Arrows indicate *gfp*-positive cells. Dotted lines indicate the outline of the gonad (Fig. 1E,F). Scale bars: 100 µm (A,C) and 10 µm (E,F,H–K). (G,L,M) Bar graphs show the percent of adult animals with extra DTCs (G), extra *mec-3::gfp*-positive cells (L), the extra *mec-4::gfp*-positive cells (M). *n*=150, 135, and 100 in control of panel G, in *ceh-22* RNAi of panel G, and in panels L and M, respectively; ***P<0.005.

We previously showed that, in *C. elegans*, malfunction of the machinery that maintains cell fate induces the production of extra distal tip cells (DTCs) (Shibata et al., 2010). In wild-type animals, there are two DTCs that function as leader cells during gonad formation. To identify additional genes that are required for the maintenance of cell fate, we screened for mutants that have extra DTCs and isolated two mutants of *tlk-1* (Fig. 1A–D; Fig. S1A). *tlk-1* encodes a serine/threonine kinase that is a member of the TLK family (Fig. S1B–E). Although humans and mice have two TLK family proteins, TLK-1 is the sole family member in *C. elegans*. *tlk-1*(*tk158)* has a nonsense mutation at Q44stop, and *tlk-1*(*tk170)* has a missense mutation at T846I (Fig. S1D). The translational termination near the N terminus suggests that *tk158* is a null allele. A DNA fragment containing the coding region and 3.5 kb of upstream sequence fully rescued the *tlk-1(tk158)* mutant phenotype (Fig. S1A). A kinase-inactive version of TLK-1 (S634A) could not rescue the *tlk-1* mutant phenotype (Fig. S1A). *tlk-1::gfp* expression was observed in the nuclei of all somatic cells including cells of the somatic gonad, neurons in the posterior lateral ganglia (PLG), and the hypodermis (Fig. S1F−I). TLK-1 is also expressed in the nuclei of embryos (Han et al., 2003). The knockdown of *tlk-1* by feeding RNAi resulted in embryonic lethality (data not shown). However, we observed the postembryonic extra-DTC phenotype in *tlk-1* homozygous mutants from heterozygous mutant hermaphrodites because the embryonic lethality was rescued by the maternal effect.

### TLK-1 functions in multiple cell lineages

In the wild-type somatic gonad, the two DTCs express *lag-2::gfp* (Kostić et al., 2003). Extra DTCs were observed in half of the *tk158* mutants (Fig. S1A). The maximum number of DTCs was five cells in *tk158* mutants. In addition to expressing *lag-2::gfp*, DTCs were positioned at the tip of the gonad arms and showed a cup-like shape in wild-type animals (Fig. 1E; Fig. S2A). *tlk-1* mutants had extra DTCs at the tips of the extra gonad arms. The extra DTCs were also cup shaped (Fig. 1F), suggesting differentiation into DTCs rather than simple ectopic expression of *lag-2::gfp*. We examined whether extra DTC formation depended on the NK-2 family homeodomain DNA-binding TF CEH-22, which induces DTCs (Table 1) (Shibata et al., 2014). Because *ceh-22* is required for the production of mother cells of DTCs (Lam et al., 2006), we performed partial knockdown of *ceh-22* by feeding RNAi. *ceh-22* RNAi in *tlk-1* mutants partially suppressed the extra-DTC phenotype (Fig. 1G). Therefore, induction of extra-DTCs in *tlk-1* mutants is dependent on *ceh-22*.

**Table 1.**
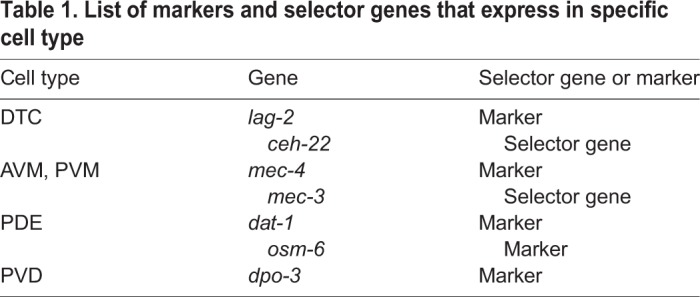
**List of markers and selector genes that express in specific cell type**

We previously reported that, in addition to the extra-DTC phenotype, malfunction of the machinery that maintains cell fate induces ectopic expression of PDE marker, PVD marker, and AVM and PVM markers (Table 1) (Shibata et al., 2010). Therefore, we next examined whether *tlk-1* functions in other cell types. *dat-1::gfp* and *dop-3::rfp* are expressed in bilateral pairs of PDE and PVD neurons, respectively, in the wild-type PLG (Chase et al., 2004; Nass et al., 2002). *dat-1::gfp* and *dop-3::rfp* markers were ectopically expressed in *tlk-1* mutants in the PLG region (Fig. S2D–E,I). We rarely observed cells with both markers; only 3% of *tlk-1* mutants exhibited this phenotype (Fig. S2F–H). In addition to gene expression, we used cell size as a characteristic of cell type. In wild type, PVD (*dop-3::rfp* positive) is larger than other neuronal cells including PDE (*dat-1::gfp* positive) (Fig. S2D). *tlk-1* mutants have multiple *dat-1::gfp*-positive cells and/or *dop-3::rfp*-positive cells, although the number of these cells varies among individuals. As observed in wild-type animals, *dop-3::rfp*-positive cells were larger than *dat-1::gfp*-positive cells in *tlk-1* mutants (Fig. S2E). Ectopic expression of another PDE marker, *osm-6::gfp*, was also observed in *tlk-1* mutants (Fig. S2I). These results suggested that ectopic PDE-like and PVD-like cells were produced in *tlk-1* mutants.

*mec-4::gfp* is expressed in AVM and PVM neurons at the anterior right and posterior left sides, respectively, in wild-type animals (Clark and Chiu, 2003) Ectopic expression of *mec-4::gfp* was observed in the region where wild-type marker-positive cells were observed (Fig. 1H,I; Fig. S2I). In wild-type animals, AVM cells expressed *mec-3* in addition to *mec-4* (Fig. 1J). We found ectopic expression of *mec-3::gfp* in *tlk-1* mutants (Fig. 1K,L). *mec-3* encodes a LIM homeodomain DNA-binding TF that induces six mechanosensory neurons including AVM (Way and Chalfie, 1988). *mec-4* is a direct target of MEC-3 (Hobert, 2008; Duggan et al., 1998). If *tlk-1* controls the AVM fate, *mec-4* expression could be regulated by *mec-3*. We examined *mec-4::gfp* expression in *tlk-1 mec-3* double mutants and found no *mec-4::gfp* expression, including its typical expression in AVM and PVM neurons and ectopic expression (Fig. 1M), suggesting that inappropriate expression of *mec-3* induces ectopic expression of *mec-4::gfp* in *tlk-1* mutants. These results suggested that TLK-1 negatively regulates genes that encode cell type-specific TFs. Yet downstream genes are regulated through cell type-specific TFs even in *tlk-1* mutants.

### Defects in cell-fate maintenance in *tlk-1* mutants

In wild-type animals, distal granddaughters of the Z1/Z4 cells that are born at the L1 stage differentiate into DTCs until the early L2 stage (Shibata et al., 2010). In *tlk-1* mutants, we compared the extra-DTC phenotype at the L2 and adult stages and found a lower penetrance at the L2 stage (Fig. S3A). Analysis of the DTC numbers in the L2 and adult stages in the same animals revealed that they increased in 12 of 29 *tlk-1* mutant animals (data not shown). In addition, we never observed cell division of normal DTCs even in *tlk-1* mutants. These results indicated that extra DTCs are produced even after the production of normal DTCs.

**Fig. 2.**
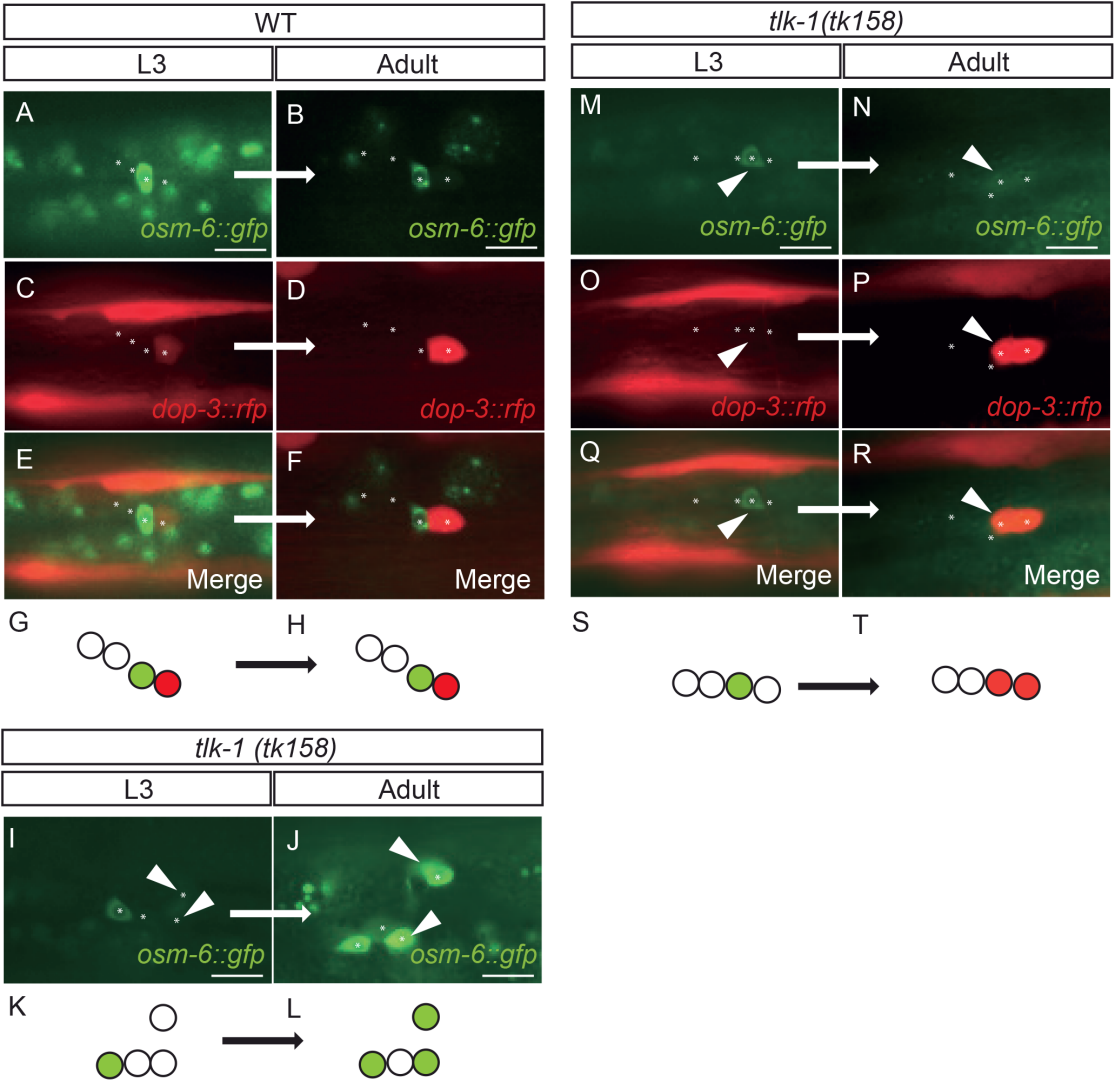
**Defects in cell-fate maintenance in *tlk-1* mutants.** Fluorescence images of wild type (WT) (A–F) and two individual *tlk-1* mutant animals (I,J,M–R). Expression was observed at the L3 stage and then at the adult stage in the same animal. Asterisks indicate the positions of neuronal nuclei detected in the DIC images. Arrowheads indicate the cells with altered marker expression between the two stages. (I,J) Some *osm-6::gfp*-negative cells at the L3 stage later expressed *osm-6::gfp* until the adult stage. (M–R) Some *osm-6::gfp*-expressing cells at the L3 stage lost this expression at the adult stage, and the same cells expressed *dop-3::rfp* at the adult stage. Schematics of each PLG are shown in G,H,K,L,S, and T. Circles represent neural cells in PLG. Green and red indicate *osm-6::gfp* and *dpo-3::rfp* expression, respectively. Anterior is to the left, ventral is to the bottom. Scale bars: 10 µm.

To elucidate the cause of extra marker-positive cells, we observed the PLG using the PDE marker *osm-6::gfp* and the PVD marker *dop-3::rfp* at the third larval stage (L3) and adult stages in the same animals. The position of the cells is variable in each animal, but the relative position of cells is conserved in the same animal during development (Shibata et al., 2010). In wild-type animals, cells that expressed *osm-6::gfp* at the L3 stage never expressed *dop-3::rfp* at the adult stage and vice versa (Fig. 2A–H). Transition of cell-specific markers in the PLG between the L3 stage and the adult stage was not observed in wild-type animals (*n*=13). In contrast, we found that *osm-6::gfp*-negative cells at the L3 stage (21.5 h after hatching) started expressing *osm-6::gfp* until the adult stage in 2 of 13 *tlk-1* mutants (Fig. 2I–L). In addition, in 3 of 13 *tlk-1* mutants, *osm-6::gfp*-expressing cells at the L3 stage no longer expressed this marker at the adult stage, and the same cell expressed *dop-3::rfp* (Fig. 2M−T), indicating that the transition from the PDE marker positive cell to the PVD marker positive cell occurred in these cells. We also observed the same *tlk-1* mutant animals at the L3 (24 h after hatching) and adult stages. Of 25 *tlk-1* mutants, two expressed both *osm-6::gfp* and *dop-3::rfp* in the same cells at the L3 stage, but these *osm-6::gfp* and *dop-3::rfp* double-positive cells became *dop-3::rfp* single-positive cells at the adult stage (Fig. S3B). Thus, transition from the PDE-marker positive cells to the PVD-marker positive cells occurred at least at the L3 stage. These results indicate that TLK-1 maintains cell fate in multiple cell lineages.

### Disruption of histone chaperone CAF1 causes a *tlk-1*-like phenotype

**Fig. 3.**
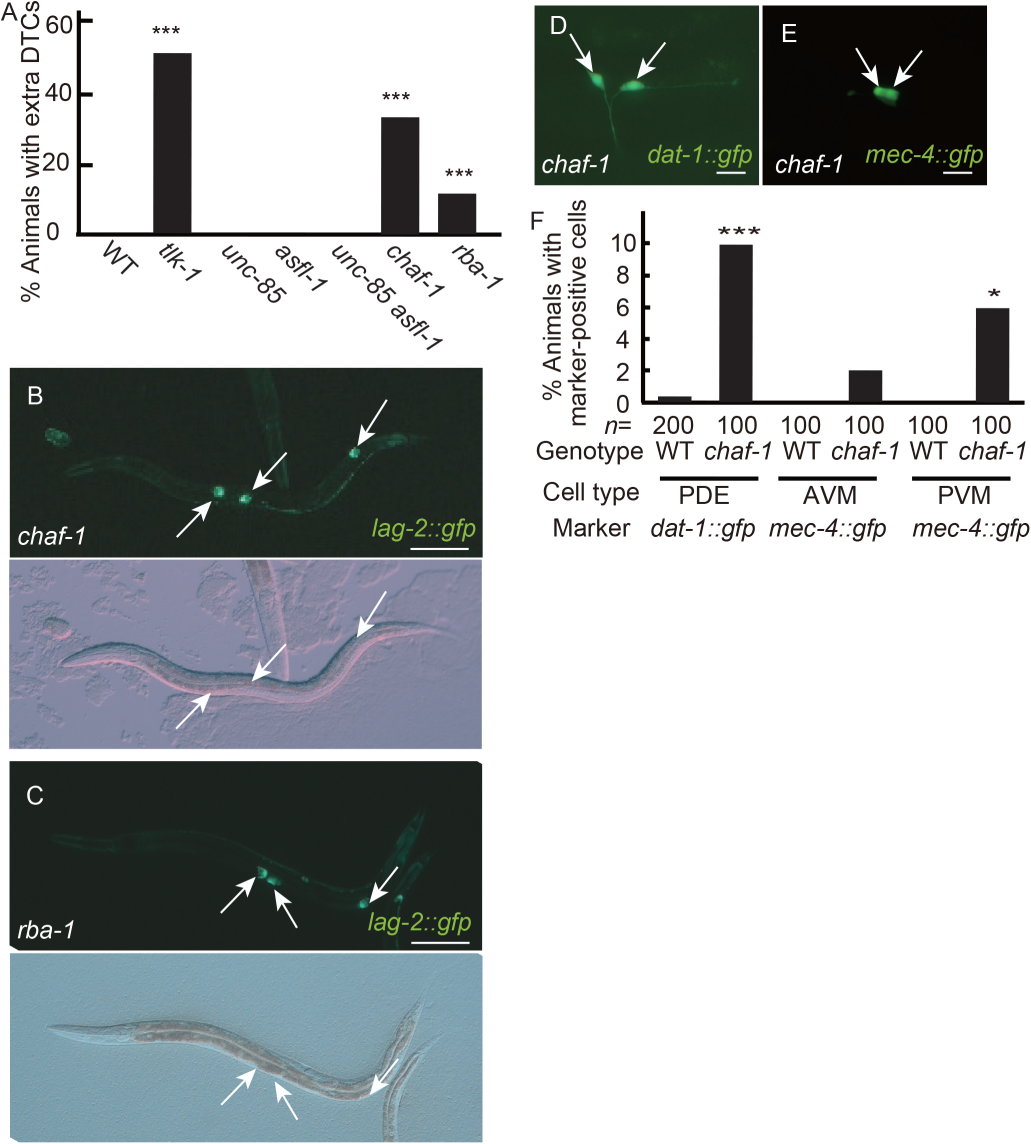
**Disruption of the CAF1 complex mimics *tlk-1* mutation.** (A,F) Bar graphs shows the percent of adult animals with the extra-DTC phenotype (A) and with *dat-1::gfp*-positive or *mec-4::gfp*-positive cells (F). (A) *n*=100; *0.05>*P*≥0.01, ***P<0.005, as compared with the wild type. (B–E) GFP images showing the expression of *lag-2::gfp* (B,C), *dat-1::gfp* (D), and *mec-4::gfp* (E) in *chaf-1* (B,D,E) or *rba-1* mutants (C) at the adult stage. Arrows indicate marker-positive cells. Scale bars: 100 µm in B and C, and 10 µm in D and E.

TLK phosphorylates ASF1 to promote histone supply to the CAF1 complex, which deposits the histone H3-H4 complex as a DNA replication-coupled histone chaperone (Klimovskaia et al., 2014). *C. elegans* has two ASF1 homologs, UNC-85 and ASFL-1 (Grigsby et al., 2009; Grigsby and Finger, 2008), but neither *unc-85* nor *asfl-1* mutants show the extra-DTC phenotype (Fig. 3A). In addition, *unc-85 asfl-1* double-homozygous mutants from *unc-85* homozygous and *asfl-1* heterozygous (*asfl-1/hT2; unc-85*) hermaphrodites did not show the extra-DTC phenotype. *unc-85 asfl-1* double-homozygous mutants from *unc-85 asfl-1* double-homozygous mutants were embryonic lethal. Thus, there was no evidence to show the requirement of ASF1 homologs in cell-fate maintenance.

*C. elegans* counterparts of CAF1 subunits are CHAF-1, CHAF-2, and RBA-1 (Nakano et al., 2011). We examined *chaf-1* mutants and *rba-1* mutants and found that they exhibited the extra-DTC phenotype (Fig. 3A–C). We also examined *dat-1::gfp* and *mec-4::gfp* expression in *chaf-1* mutants. As in *tlk-1* mutants, extra *dat-1::gfp*-positive cells and extra *mec-4::gfp*-positive cells were observed (Fig. 3D–F). Thus, the CAF1 deficiency caused phenotypes similar to those observed in *tlk-1* mutants. These results suggested that *chaf-1* functions in the same genetic pathway with *tlk-1*.

### Nuclear H3.3 levels are upregulated in *tlk-1* mutants

**Fig. 4.**
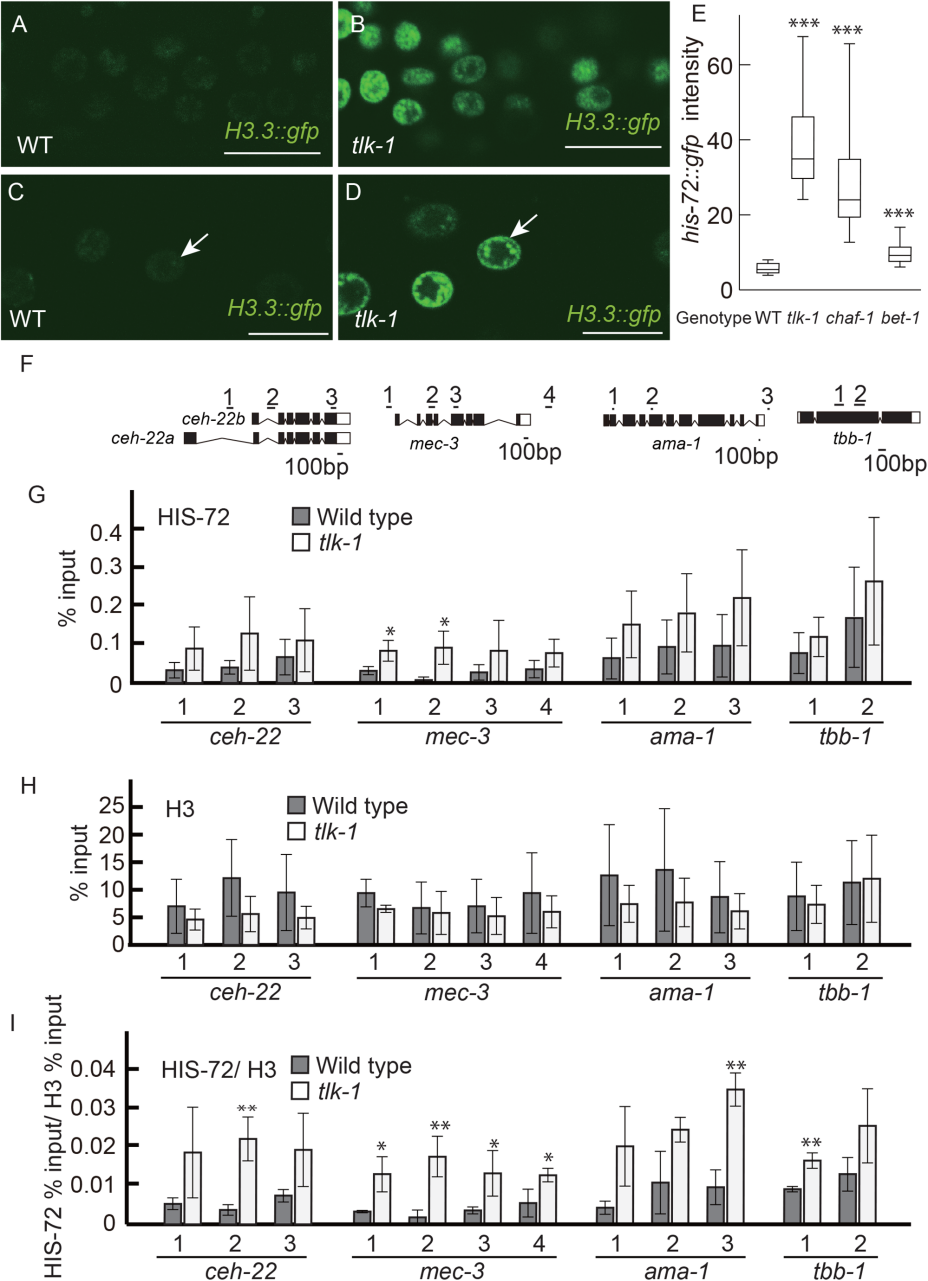
***tlk-1* negatively regulates the deposition of HIS-72/ H3.3.** (A–D) GFP images of gonadal cells (A,B) and V5.ppp cells (C,D) in wild type (WT) (A,C) and *tlk-1* mutants (B,D) at the L3 stage. Arrows indicate the nucleus of V5.ppp. All images were captured by confocal microscopy at the same setting. Scale bars: 10 µm. (E) Box-and-whisker plot showing the fluorescence intensity of HIS-72::GFP in the nucleus. *n*=20. Whiskers indicate the 10th and 90th percentiles. Boxplots represent the medians and the 25th–75th percentile. (F) ChIP assays were analyzed by qPCR using primers for DNA segments corresponding to the *ceh-22*, *mec-3*, *ama-1*, and *tbb-1* loci as shown. Open boxes and closed boxes indicate non-coding and coding exons, respectively. (G,H) The bar graphs show the percent immunoprecipitation (%IP) of each PCR fragment relative to input DNA control. (I) The bar graph shows the ratio between the percentage of total input DNA for HIS-72 and H3 ChIP samples. (G–I) Data represent the average of three independent ChIP experiments±s.d.

In cultured cells, CAF1 depletion causes alternative deposition of H3.3 that is observed on actively transcribed loci (Wirbelauer et al., 2005; Ray-Gallet et al., 2011). Although *C. elegans* has five H3.3 genes, only *his-71* and *his-72* are expressed in somatic cells (Delaney et al., 2018). In addition, the expression of *his-72* is higher than that of *his-71*. To examine the level of H3.3 deposition onto chromatin in *chaf-1* and *tlk-1* mutants, we observed the expression of HIS-72 (Ooi et al., 2006; Boeck et al., 2011) using a translation GFP fusion gene. The granular pattern of HIS-72::GFP expression in the nucleus suggested that HIS-72::GFP was deposited on the chromatin. In all observed cells, including in the somatic gonadal cells, higher HIS-72::GFP expression was detected in *tlk-1* mutants than in wild-type animals (Fig. 4A–D).

We also quantified the fluorescence intensity of HIS-72::GFP in the nucleus. Because identification of the hypodermal V5.ppp cell was possible, even in *tlk-1* and *chaf-1* mutants, we chose V5.ppp to quantify the level of HIS-72. Higher HIS-72::GFP expression was observed in the nuclei of the hypodermis of *tlk-1* and *chaf-1* mutants than in the wild-type animals (Fig. 4E). The expression level was higher in *tlk-1* mutants than in *chaf-1* mutants, which was consistent with a higher penetrance of the extra-DTC phenotype in *tlk-1* mutants (Fig. 3A). In contrast to *tlk-1* mutants, mutants of *bet-1*, which functions through the deposition of H2A.z (Shibata et al., 2014), showed only weak upregulation of HIS-72::GFP expression (Fig. 4E). We also analyzed H2B::mCherry fluorescence and found no significant difference between wild-type animals and *tlk-1* mutants (Fig. S4A). These results strongly suggested that TLK-1 and CAF1 reduce HIS-72::GFP accumulation in chromatin.

Our analyses of mutants suggested that *mec-3* and *ceh-22* play important roles in cell-fate maintenance. These gene loci may be direct targets of HIS-72 in *tlk-1* mutants. To examine genomic localization of HIS-72, we performed chromatin immunoprecipitation (ChIP) by using hemagglutinin (HA)-tagged HIS-72. We also analyzed two loci for housekeeping genes, *ama-1*, which encodes RNA polymerase II, and *tbb-1*, which encodes β-tubulin in addition to *ceh-22* and *mec-3* (Fig. 4F). In the wild-type background, HIS-72 tended to be more enriched on the *ama-1* and *tbb-1* loci than on the *ceh-22* and *mec-3* loci, consistent with the earlier finding that HIS-72 abundance correlates with the level of gene expression (Ooi et al., 2010) (Fig. 4G; Fig. S5). A significant increase in HIS-72 deposition was observed only on the *mec-3* locus, indicating that the effect of *tlk-1* depletion is more pronounced on the *mec-3* locus. We also performed a ChIP assay by using anti-H3, which recognizes all the histone H3 isoforms, and found no significant difference between the wild-type and the *tlk-1* background at all four loci (Fig. 4H), indicating that there is no significant reduction in nucleosomes in *tlk-1* mutants. We also analyzed the ratio between HIS-72 and H3 (Fig. 4I). At least one of the positions examined showed significant increase of HIS-72 accumulation in the *tlk-1* background in *ceh-22*, *ama-1* and *tbb-1* loci, and all the positions showed significantly higher HIS-72 levels in the *mec-3* locus with the *tlk-1* background. These results suggest that the levels of H3.3 accumulation are upregulated in these four loci, and that the upregulation is especially prominent in *mec-3* in the *tlk-1* background (Fig. 4F,I). In wild-type animals, *mec-3* is expressed in ten neurons including AVM and PVM (Way and Chalfie, 1989). In contrast, *ceh-22* is expressed in multiple tissues, including the pharynx, intestine, and neurons in the head, tail, and ventral nerve cord (Lam et al., 2006). Because *mec-3* is expressed in a smaller number of cells than does *ceh-22*, we expected that there would be lesser noise for the *mec-3* locus from the ChIP assay compared with the *ceh-22* locus.

### Regulation of H3.3 by HIRA-1

In mammals, TLK-1-dependent phosphorylation of ASF1 enhances ASF binding to histones and to the chaperones CAF-1 and HIRA. Mammalian HIRA is known as a chaperone of H3.3 (Tagami et al., 2004). These facts suggest that malfunction of TLK may disturb H3.3 deposition in addition to H3 deposition. In contrast, our results showed that H3.3 does accumulate in *tlk-1* mutants. This inconsistency prompted us to investigate the relationship between *tlk-1* and *hira-1* in *C. elegans*. Interestingly, although *hira-1* single mutants did not show the extra-DTC phenotype, upregulation of HIS-72::GFP fluorescence was observed (Fig. S4B,C). Furthermore, *hira-1* deletion enhanced the extra-DTC phenotype and upregulation of HIS-72::GFP fluorescence in the *tlk-1* background (Fig. S4B–D). Although HIRA-1 is known to function as an H3.3 chaperone, a HIRA-1-independent mechanism appears to promote H3.3 deposition in the *tlk-1* background in *C. elegans*.

### Loss of SIN-3 suppresses extra-DTC phenotype of *tlk-1* mutants

**Fig. 5.**
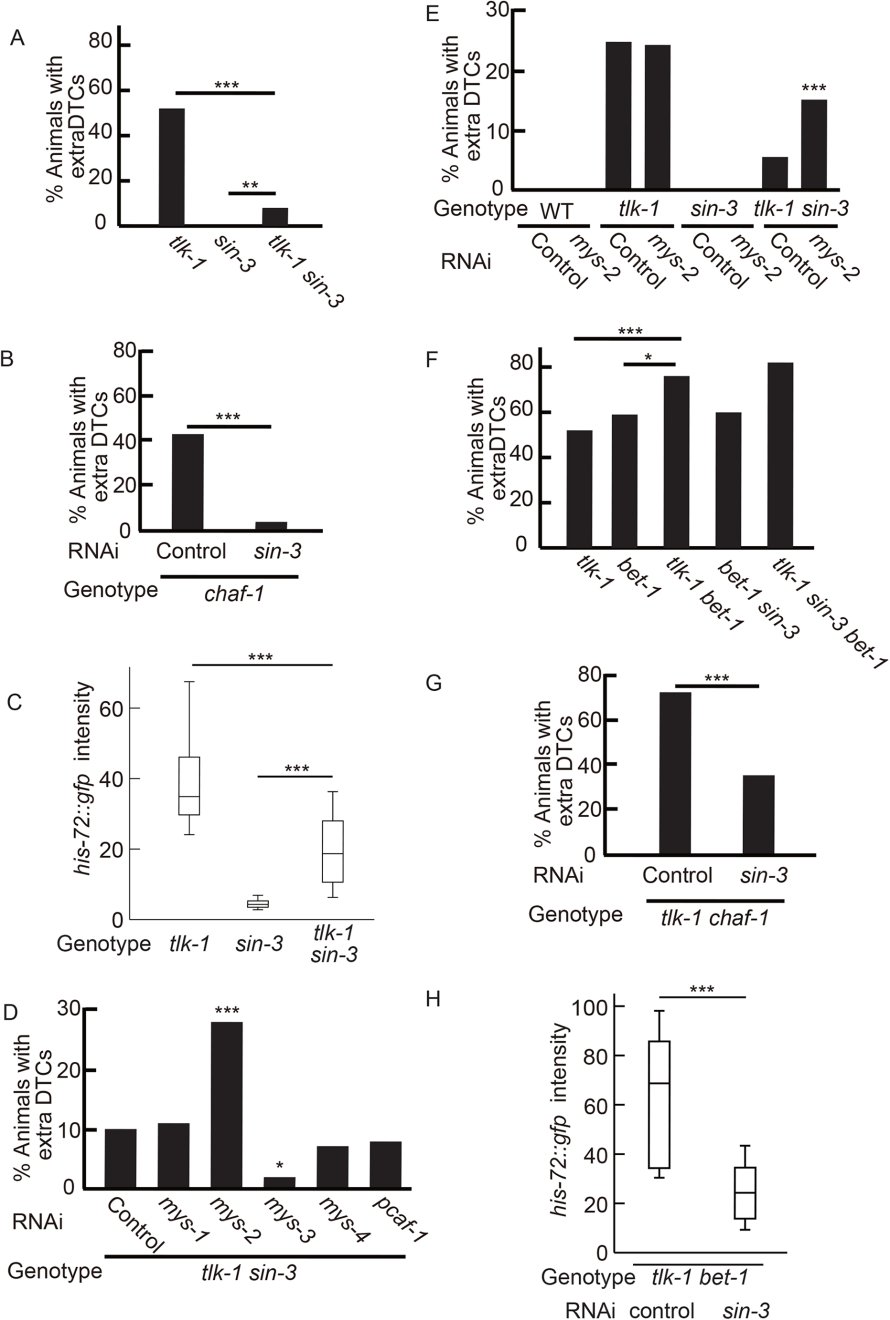
***sin-3* as a suppressor of *tlk-1*.** (A,B,D–G) Bar graphs show the percent of adult animals with extra DTCs. *n*=100. (C,H) Box-and-whisker plot showing the fluorescence intensity of HIS-72::GFP in the nucleus. *n*=20. Whiskers indicate the 10th and 90th percentiles. Boxplots represent the medians and the 25th–75th percentile. *, **, and *** indicates 0.05>*P*≥0.01, 0.01>*P*≥0.005, *P*<0.005, respectively.

We performed RNAi screening for suppressors of *tlk-1* mutants using the chromatin subset of the Ahringer's feeding RNAi library (Kamath et al., 2003). We found that *sin-3* RNAi suppressed the extra-DTC phenotype of *tlk-1* mutants (Fig. S6A). A *sin-3* deletion mutant also suppressed the *tlk-1* phenotype (Fig. 5A). Most *sin-3 tlk-1* double mutants had two DTCs (Fig. S6B). The frequency of animals with one or no DTCs was similar between the double mutants and the s*in-3* single mutants. Of note, *sin-3* RNAi also suppressed the extra-DTC phenotype of *chaf-1* mutants (Fig. 5B). Thus, *sin-3* was antagonistic to *tlk-1* rather than having a role in DTC differentiation. We also examined the expression of HIS-72::GFP in the *tlk-1 sin-3* background. Interestingly, the level of HIS-72::GFP expression decreased in *tlk-1 sin-3* relative to its expression in *tlk-1* (Fig. 5C).

### Acetylated histone-binding protein BET-1 is necessary for suppression by *sin-3* disruption

The SIN3 complex contains histone deacetylase, HDAC (Hassig et al., 1997; Laherty et al., 1997). However, HDAC (*had-1*, *2*, *3*, *4* and *6*) RNAi did not suppress the extra-DTC phenotype in *tlk-1* mutants (Fig. S6C). If histone hyper-acetylation in *tlk-1 sin-3* double mutants is responsible for suppression of the extra-DTC phenotype, disruption of histone acetyltransferase may induce extra DTCs. We found that RNAi of *mys-2*, which encodes MYST family histone acetyltransferase, induced the extra-DTC phenotype in the *tlk-1 sin-3* background (Fig. 5D). *mys-2* RNAi did not enhance the extra-DTC phenotype in the *tlk-1* background and did not induce extra DTCs in the wild-type or *sin-3* background (Fig. 5E). Therefore, the effect of *mys-2* RNAi was specific to the *tlk-1 sin-3* background. These results suggested that the extra-DTC phenotype in *tlk-1* mutants can be compensated for by hyper-acetylation.

MYS-2 controls sub-nuclear localization of the acetylated histone H4 binding protein BET-1 (Shibata et al., 2014, 2010). Because we originally isolated *tlk-1* mutants as a phenocopy of *bet-1* mutants, we examined the relationship between *tlk-1* and *bet-1*. First, we examined the phenotype of *tlk-1 bet-1* double mutants. We found that *tlk-1 (tk158)* enhanced the *bet-1* null allele, *os46* (Fig. 5F). The maximum number of DTCs was seven in *tlk-1 bet-1* double mutants, in contrast to four and five in *bet-1* and *tlk-1* mutants, respectively. Therefore, *tlk-1* acts in parallel with *bet-1* to maintain the DTC cell fate. We also found that the *tlk-1* suppressor *sin-3* could not suppress *bet-1* (Fig. 5F). These results are consistent with parallel regulation by *bet-1* and *tlk-1*.

If SIN-3 regulates BET-1 through acetylation, *sin-3* suppression should be dependent on *bet-1*. However, if SIN-3 and BET-1 control parallel pathways, *sin-3* should suppress the extra-DTC phenotype of *tlk-1* mutants even without *bet-1*. The results revealed that *sin-3* did not suppress the extra-DTC phenotype of *tlk-1 bet-1* double mutants, indicating that the *sin-3* suppression was dependent on *bet-1* (Fig. 5F). Although the phenotypic penetrance of *tlk-1 chaf-1* was similar to that of *tlk-1 bet-1*, *sin-3* RNAi suppressed the extra-DTC phenotype only in the *tlk-1 chaf-1* background (Fig. 5G). These results indicated that *bet-1* is downstream of *sin-3*.

Next, we examined whether SIN-3 regulates the level of H3.3 deposition through BET-1. *sin-3* RNAi suppressed the level of HIS-72::GFP expression in the *tlk-1 bet-1* background (Fig. 5H), indicating that BET-1 is not required for the suppression of H3.3 accumulation by *sin-3* depletion in *tlk-1* mutants.

## DISCUSSION

### TLK-1 and CAF1 maintain cell fates

In this study, we identified a gene that is required for cell-fate maintenance, *tlk-1*, which encodes a TLK family serine/threonine kinase. Transition of cell-fate markers from those of PDE to PVD indicated that *tlk-1* is required for cell-fate maintenance. We speculate that defects in cell-fate maintenance also produce extra-DTCs and extra-AVMs. Although it is known that TLK is required for development in *C. elegans* and Arabidopsis (Roe et al., 1993; Wang et al., 2007; Han et al., 2003, 2005), this is the first report demonstrating the relationship between TLK and cell-fate maintenance. Biochemical analyses using mammalian cell lines revealed that TLK1 acts upstream of the CAF1 complex (Klimovskaia et al., 2014). The *C. elegans* CAF1 complex is required in development for the regulation of the bilateral asymmetric cell lineage (Nakano et al., 2011). The relationship between TLK and the CAF1 complex in development has not, however, been studied. Our results that showed a phenotypic similarity between *tlk-1* and *chaf-1* mutants support the importance of the relationship between TLK and the CAF1 complex in cell-fate maintenance. Here, we showed that TLK-1 and the CAF1 complex are required for cell-fate maintenance through preventing ectopic expression of genes that encodes DNA-binding TFs.

### TLK-1 and CAF1 appear to regulate histone H3.3 in cell-fate maintenance

The CAF1 complex deposits the histone H3-H4 complex as a DNA replication-coupled histone chaperone (Klimovskaia et al., 2014). In mammalian cell lines impaired H3 incorporation by CAF1 depletion causes alternative deposition of H3.3 (Ray-Gallet et al., 2011). We revealed that, in *C. elegans*, depletion of TLK-1 or the CAF1 complex upregulates nuclear H3.3 levels. These findings indicated that the alternative deposition of H3.3 in the CAF1-deficient background appears to be evolutionarily conserved. H3.3 is localized to actively transcribed loci (Wirbelauer et al., 2005). An important question is whether CAF1 maintains cell fate by preventing the alternative deposition of H3.3. Interestingly, our results indicated that the level of H3.3 accumulation and the extra-DTC phenotype of *tlk-1*-related mutants (*tlk-1*, *chaf-1*, *rba-1*, *tlk-1 sin-3*, *tlk-1 hira-1*) are highly correlated. Analyses of mutants showed that the repression of selector genes, for example *mec-3* and *ceh-22*, is important for cell-fate maintenance. Enrichment of H3.3 on the *mec-3* locus in *tlk-1* mutants is consistent with our hypothesis that TLK-1 and the CAF1 complex maintain cell fate though the regulation of H3.3 deposition. Another possible cause of ectopic gene expression is the reduction or loss of nucleosomes. However, we detected no significant reduction in nucleosomes. Based on the correlation between the extra-DTC phenotype and nuclear H3.3 levels, we propose the hypothesis that H3.3 enrichment on selector gene loci contributes to the activation of transcription.

In addition to the *mec-3* locus, the level of H3.3 in *tlk-1* mutants tends to increase on the loci of the housekeeping genes *ama-1* and *tbb-1,* relative to wild-type animals (Fig. 4G). Together with upregulation of nuclear H3.3 (Fig. 4E), it is possible that the alternative deposition of H3.3 occurs on many loci in *tlk-1* or *chaf-1* mutants. The significant H3.3 enrichment on the *mec-3* locus in *tlk-1* mutants based on whole-animal ChIP analysis suggested that H3.3 accumulation in *mec-3* occurs in a relatively large number of cells. However, the fate maintenance-defective phenotype of *tlk-1* mutants was detected in specific cell types. We speculate that the deposition of H3 by the CAF1 complex on selector gene loci is important for repressing their transcription. Selector genes can be activated only in specific cell types, probably through H3.3 deposition, in the absence of TLK-1. Presumably, additional silencing mechanisms prevent other cells from stochastic transcriptional activation of selector genes. The feed-forward loop to promote the expression of a selector gene (Hobert, 2008) may amplify the effect of stochastic transcriptional activation that is caused by the alternative deposition of H3.3.

### TLK-1 and BET-1 act in parallel pathways

We found that loss of SIN-3 strongly suppressed the extra-DTC phenotype of *tlk-1* and *chaf-1* mutants. The SIN3 complex contains HDAC (Hassig et al., 1997; Laherty et al., 1997), suggesting that the acetylation of histones is elevated in *sin-3* mutants. Although we could not find a HDAC that acts in cell-fate maintenance, RNAi knockdown of one of the histone acetyl transferases, *mys-2*, reversed the suppression effect of *sin-3* mutants. This result suggested that SIN-3 acts though acetylation in the regulation of cell-fate maintenance. Because depletion of *sin-3* downregulated H3.3, it is likely that SIN-3 is a part of the mechanism that promotes H3.3 deposition.

The suppressor activity of *sin-3* mutants depended on an acetylated histone-binding protein, BET-1, which is required to maintain the fate of cell types whose fates are also maintained by TLK-1 and CHAF-1 (Shibata et al., 2010). Because the cell fate-maintenance defect was enhanced in *tlk-1* and *bet-1* double mutants, TLK-1 and BET-1 act in distinct pathways to regulate cell-fate maintenance.

### Model for cell-fate maintenance by histone variants

**Fig. 6.**
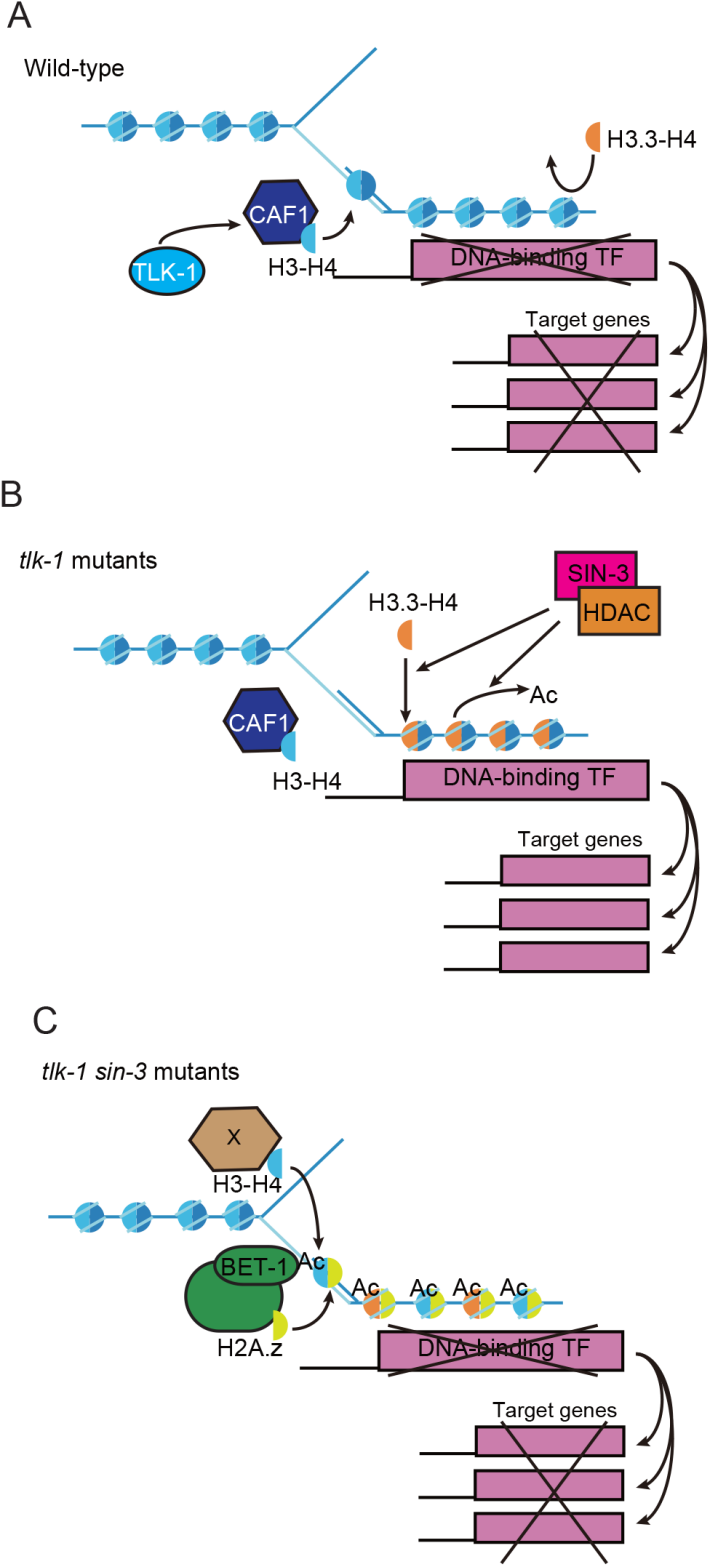
**Model for cell-fate maintenance.** (A) In wild-type animals, TLK-1 and CAF1 promote formation of H3-containing nucleosomes that prevent alternative deposition of H3.3. (B) In *tlk-1* mutants, H3.3 is incorporated into the nucleosome-free region that is formed by dysfunction of the CAF1 complex. H3.3 causes stochastic expression of DNA-binding TFs. (C) There are fewer H3.3-containing nucleosomes in *tlk-1 sin-3* double mutants than in *tlk-1* mutants. Loss of *sin-3* causes hyper-acetylation, which, most likely, recruits BET-1. We speculate that lower H3.3 and higher BET-1 contributes to transcriptional repression.

Based on our findings, we propose the following model. Localization of histone H3 on selector gene loci is critical for transcriptional repression (Fig. 6A). H3 deposition by TLK-1 and CAF1 inhibits deposition of H3.3. In *tlk-1* mutants, reduction of CAF1 activity causes ectopic H3.3 deposition (Fig. 6B). Stochastic expression of selector genes by ectopic H3.3 deposition causes the failure in cell-fate maintenance. The SIN-3 complex acts in H3.3 deposition as well as in histone de-acetylation. In the *tlk-1* mutant background, loss of SIN-3 causes downregulation of H3.3 and hyper-acetylation of histones (Fig. 6C). Hyper-acetylated histones may enhance accumulation of BET-1 that binds acetylated histones (Shibata et al., 2010). BET-1 acts in the recruitment of H2A.z, which is required for transcriptional repression of selector genes (Shibata et al., 2014) and thereby maintains cell fates. Although it is thought that H2A.z localization is correlated with transcriptional activation, we previously showed that H2A.z represses transcription in cell-fate maintenance (Shibata et al., 2014). These results, together with the current research, lead us to speculate that H3.3 localization is correlated with transcriptional activation, whereas H2A.z localization is correlated with transcriptional repression in cell-fate maintenance. The balance between H3.3 and H2A.z on selector gene loci may be important for the maintenance of cell fate. Biophysical evidence indicates that H2A.z promotes chromatin compaction, thereby restricting gene transcription (Chen et al., 2013). In contrast, H3.3 promotes gene activation, counteracting H2A.z-mediated transcriptional repression by impairing H2A.z-mediated chromatin compaction, thus reducing higher-order chromatin folding (Chen et al., 2013). These histone variants may co-regulate transcription through chromatin compaction in cell-fate maintenance.

Recent studies revealed that artificial induction of trans-differentiation, including generation of iPS cells, is improved by repression of CAF1 (Cheloufi et al., 2015). The current study demonstrated the role of CAF1 and TLK-1 in cell-fate maintenance during normal development. Although mammalian cases have been examined under artificial conditions, the roles of CAF1, and probably of TLK, in the maintenance of cell fate appear to be conserved in multicellular organisms.

Our research also showed that there is a strong correlation between the H3.3 level and defects in cell-fate maintenance, suggesting that the regulation of H3 variants is important for cell-fate maintenance. H3.3 and H2A.z are major histone variants that are conserved in yeast, *C. elegans*, and mammals. Crosstalk between these histone variants may be a fundamental mechanism in cell-fate maintenance. The roles of TLK, H3.3, SIN3, BET family proteins, and H2A.z remain unknown in artificial trans-differentiation. Thus, the functional conservation of TLK, CAF1, SIN3, and histone variants between cell-fate maintenance in *C. elegans* and artificial trans-differentiation in mammals is an interesting issue for future studies.

## MATERIALS AND METHODS

### Strains and culture

N2 Bristol was used as the wild-type *C. elegans* strain (Brenner, 1974). Animals were cultured at 20°C. The *bet-1* (Shibata et al., 2010), *chaf-1*, *rba-1* (Nakano et al., 2011), *hira-1* and *tlk-1* mutants are sterile and were maintained as heterozygotes over the hT2[qIs48] balancer. The phenotypes of homozygotes generated from the heterozygous hermaphrodites were analyzed. The following green fluorescent protein (GFP) and red fluorescent protein (RFP) markers were used: *zdIs5[mec-4::gfp]* (Clark and Chiu, 2003), *qIs56[lag-2::gfp]* (Kostić et al., 2003), *mnIs17[osm-6::gfp]* (Collet et al., 1998), *vsIs33[dop-3::rfp]* (Chase et al., 2004), *uIs22[mec-3::gfp]* (Toker et al., 2003), *vtIs1[dat-1::gfp]* (Nass et al., 2002), *stIs10026[his-72::gfp]* (Boeck et al., 2011), *zuIs235[his-72p:: BIOTAG::3XHA::HIS-72:]* (Ooi et al., 2010). Synchronization of animals was performed as described (Shibata et al., 2010).

### RNAi

The *sin-3*, *mys-1*, *mys-2*, *mys-3*, *mys-4*, *pcaf-1*, *hda-1*, *hda-2*, *hda-3*, *hda-4*, *hda-6*, and *ceh-22* RNAi constructs were described previously (Shibata et al., 2010; Kamath et al., 2003). Feeding RNAi experiments were performed as described previously (Kamath et al., 2001). RNAi screening was performed using the *C. elegans* RNAi chromatin library (Source BioScience, Nottingham, UK).

### Cloning of *tlk-1*

Single-nucleotide polymorphism (SNP) mapping indicated that both *tk158* and *tk170* are positioned on LG III. A complementation test revealed that *tk158* and *tk170* are allelic (data not shown). Because *tk158* showed a more severe phenotype (Fig. S1A), we used *tk158* for further analysis. *tk158* was positioned at the center cluster of LGIII between 0.92 and 1.13 by SNP mapping (Fig. S1B). Because *tk158* and *tk170* are sterile, heterozygous animals that are balanced by hT2 were used for genome sequencing. Within the candidate region, *tlk-1* is the sole gene that has a non-synonymous mutation in both *tk158* and *tk170* mutants. For the rescue experiment, a PCR fragment that contained *tlk-1* and 3.5 kb of upstream sequence was amplified from the fosmid WRM0631bB10 using primers 5′-CTCTCTTTGCCACTTTATCGTTTGT-3′ and 5′-AAGTTTGCGCATGTAGTAAGTTTCA-3′. The transgenic marker was *myo-3::mCherry*.

### Microscopy and statistical analysis

Expression of *lag-2::gfp*, *dat-1::gfp*, *osm-6::gfp*, *dop-3::rfp*, and *mec-3::gfp* was detected by epifluorescence microscopy (AxiosImagerM2 and Axioplan2; Zeiss, Jena, Germany). Expression of HIS-72::GFP was detected by confocal microscopy (LSM510 and Pascal; Zeiss) in the L3 animals. We used V5.ppp to quantify HIS-72::GFP because V5.ppp is easy to identify in wild-type animals and mutants, has a large nucleus, and is positioned near the body surface. The average intensity in the V5.ppp nucleus was measured using ImageJ (NIH). Background fluorescence was measured from the adjacent region of the nucleus of V5.ppp and subtracted from the average intensity.

### ChIP assay

ChIP assays were performed as described (Ikeda et al., 2017). Worms were sonicated in an M220 focused-ultrasonicator (Covaris Inc., USA). Immunoprecipitation was performed by using SureBeads Protein G (Bio-Rad, 161-4023). The antibodies used for immunoprecipitation were as follows: anti-H3 (Abcam, ab1791), anti-HA (Roche, 3F10), normal rabbit IgG (Abcam, ab171870), and normal rat IgG (Santa Cruz Biotechnology, SC-3882). The relative enrichment levels were measured by Quantitative PCR (qPCR) using Light Cycler Nano (Roche), FastStart Essential DNA Green Master (Roche), and the following primers: *ceh-22* 1, attcaagccttttagcgttgc and aaaatggggagaacatggttg; *ceh-22* 2, aacaccttcccgtgagaacac and gcatccattcatttccgattc; *ceh-22* 3, ctaccaacaccttccgcctac and aaggccaccattgagtattgg; *mec-3* 1, gtcaccatttggagacaccag and aacaaatcacccgtcaagagc; *mec-3* 2, tctcctctggccgaaaagtg and ccgcacaccgatgaatactg; *mec-3* 3, tttgtgtggacggcatttatc and gtgcgttgtcatccatttgag; *mec-3* 4, ttgtcaacgccttcgtgatac and tggggaaggaaagaaaaagtg; *ama-1* 1, cgttgcgtatgcttctactgc and ttggctttgcacagatcgtag; *ama-1* 2, cgtccgtatgatgacaaaacg and gacttgttttccggtccaaag; *ama-1* 3, tgacacggactagaacgatgc and gggagatgagacgcagacatc; *tbb-1* 1, ccagctcacacactctcttgg and acctttggtgatggaacaacc; *tbb-1* 2, accaacccaacatacggagac and atgaagacgtgggaatggaac. All primers shown as 5′–3′.

## Supplementary Material

Supplementary information
