# Supplementary material for: Maintenance of cell fates and regulation of the histone variant H3.3 by TLK kinase in Caenorhabditis elegans
Source: Biol Open. 2019 Jan 11;8(1):bio038448. doi: 10.1242/bio.038448 (PMC6361200; doi:10.1242/bio.038448)
Supplement: Supplementary information [file biolopen-8-038448-s1.pdf]

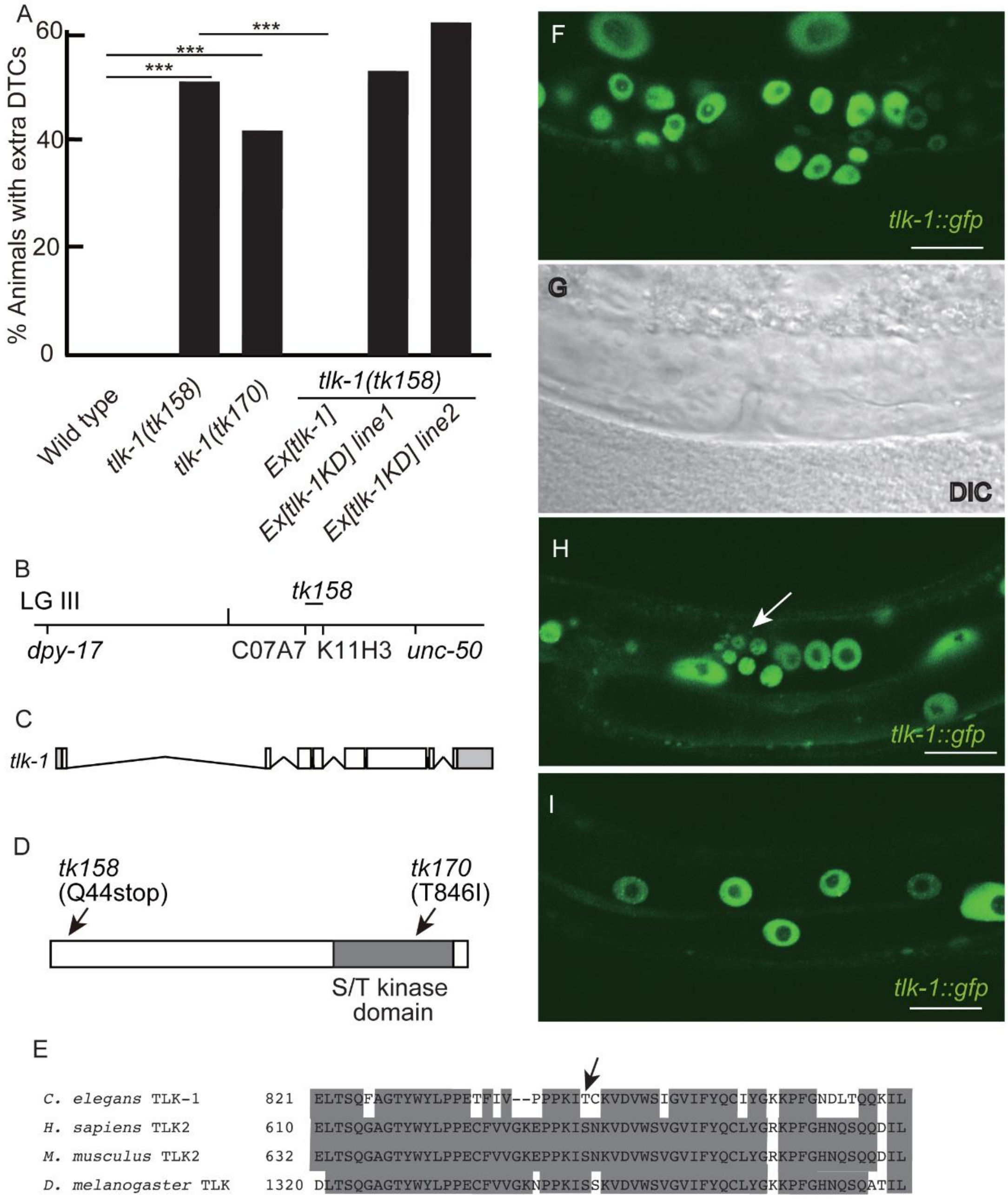

Fig. S1. Isolation of *tlk-1* mutants. (A) Bar graph showing the percent of adult animals with the extra-DTC phenotype. n = 100; ***p < 0.005, as compared with wild type. (B–D) Schematic diagrams of the chromosomal position of *tlk-1* (B), *tlk-1* gene structure (C), and TLK-1 protein structure (D). (C) Coding and non-coding regions are indicated by white and gray boxes, respectively. (D) Serine threonine

Biology Open • Supplementary information

(S/T) kinase domain is indicated by the gray box. Arrows indicate the positions of *tk158* and *tk170* mutations. (E) Alignment of TLK-1 and its homologs. Arrow indicates the position of the *tk170* mutation. Mutation site of *tk170*, T846I, is positioned in the serine/threonine kinase domain and corresponds to the serine residue in other organisms including humans, mice, and *Drosophila*. (F, H, I) *tlk-1::gfp* expression in the somatic gonad (F), PLG (H), and hypodermis (I) of L4 larvae. Arrow indicates the PLG. Scale bars indicate 10 μm. (G) DIC image that corresponds to panel (F).

Biology Open • Supplementary information

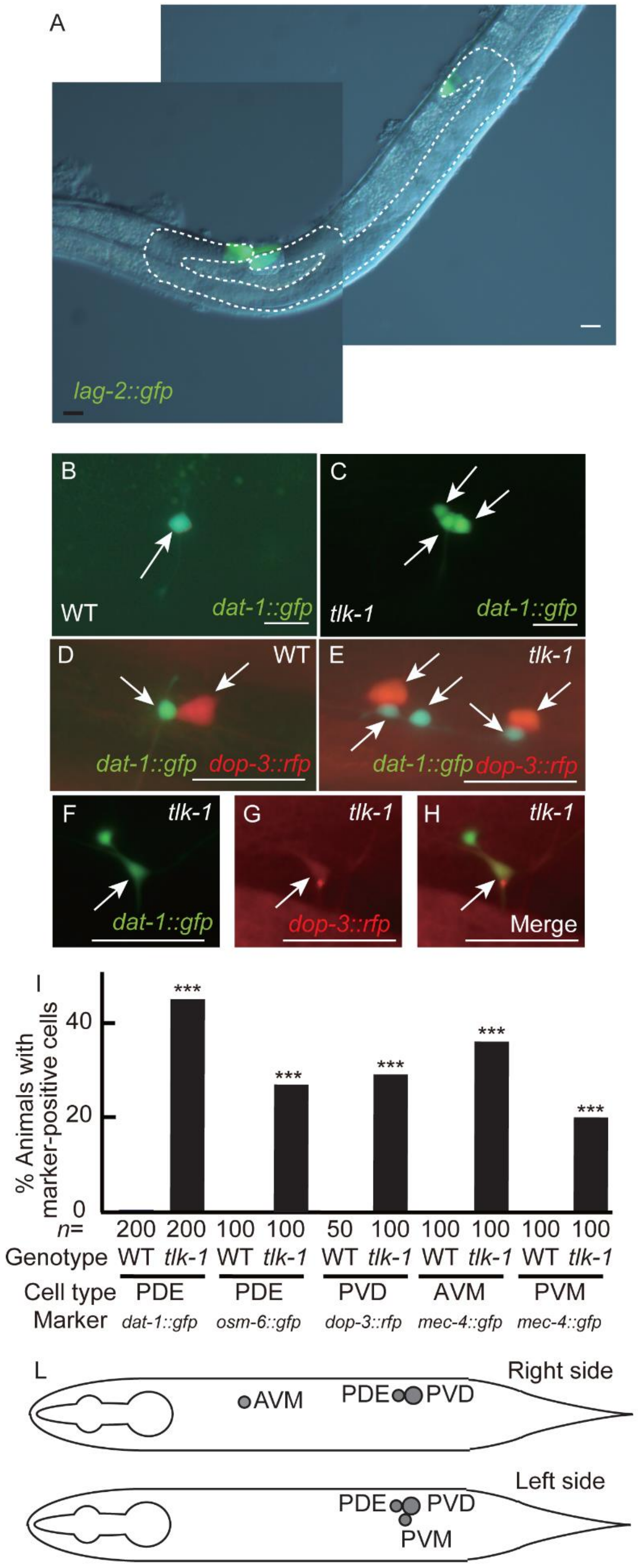

Biology Open • Supplementary information

Fig. S2. *tlk-1* functions in multiple cell types. (A) Image showing the expression of the DTC marker *lag-2::gfp*. **Dotted lines indicate the outline of the gonad**. (B–H) Fluorescence images showing the expression of markers *dat-1::gfp* (B, C, F), *dat-1::gfp* and *dop-3::rfp* (D, E, H), *dop-3::rfp* (G in wild type (WT) (B, D) and *tlk-1* mutants (C, E–H) at the adult stage. Arrows indicate marker-positive cells. Scale bars indicate 10 μm. (I) Bar graphs show the percent of adult animals with extra marker-positive cells. (L) Schematics showing the position of AVM, PVM, PDE, and PVD. Gray circles represent neural cells. The positions of the neural cells in PLG vary across animals.

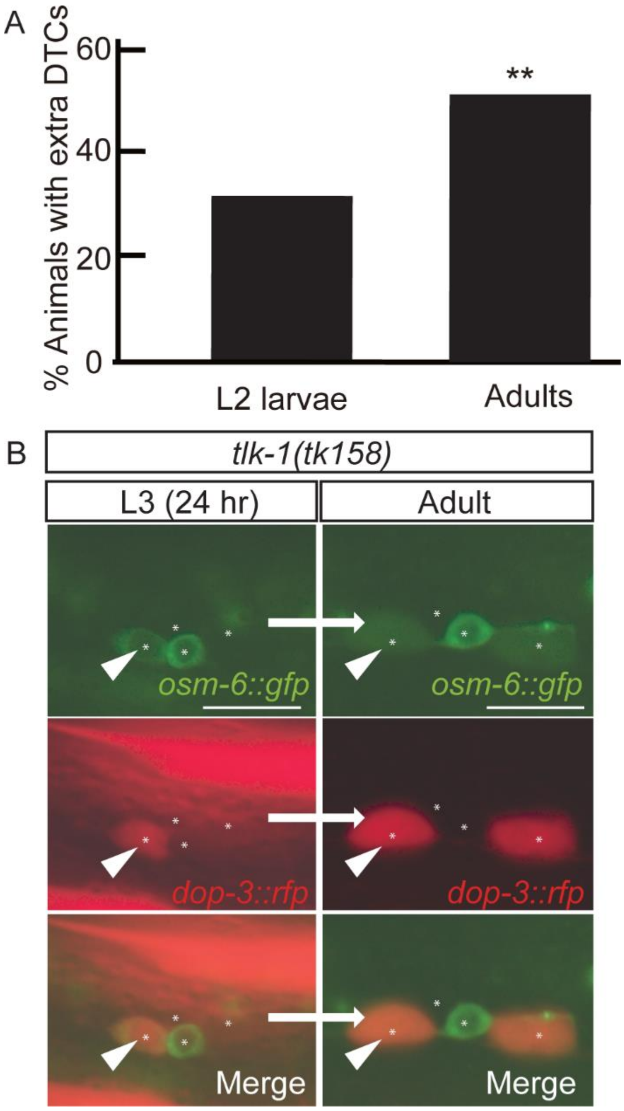

Fig. S3. Transition of cell-specific markers in *tlk-1* mutants during development. (A) Bar graph shows the percent of *tlk-1* mutants with the extra-DTC phenotype among L2 larvae and adults. n = 100; **0.005 > p ≥ 0.01, as compared with the L2 larvae. (B) Fluorescence images of individual *tlk-1* mutants. Expression of the indicated markers was observed at the L3 stage and then at the adult stage in the same animal. Asterisks indicate the positions of neuronal nuclei detected in the DIC images. Arrowheads indicate the cells with altered marker expression between the two stages. Anterior is to the left, ventral is to the bottom. Scale bar in upper image indicates 10 μm in all six images.

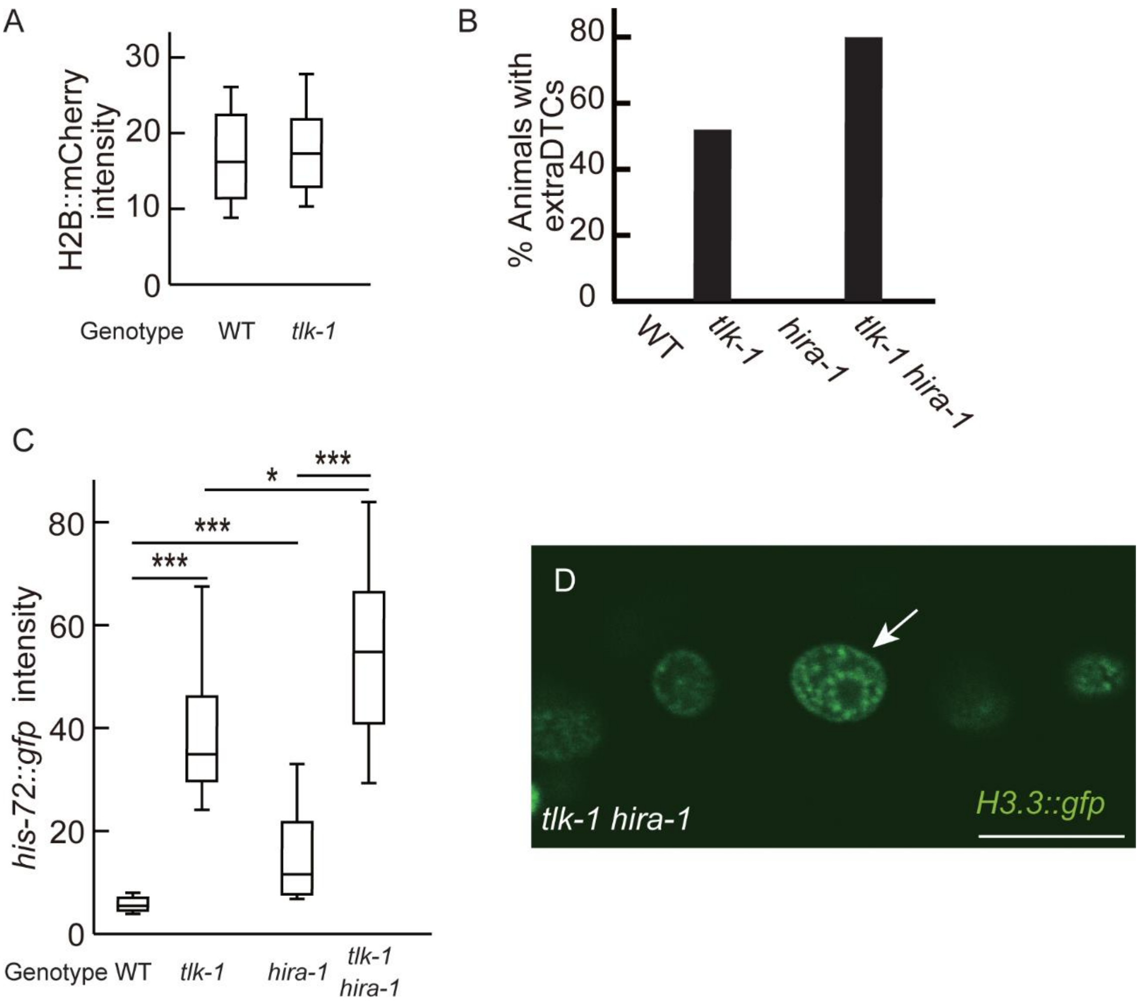

Fig. S4. Regulation of H3.3 by HIRA-1. (A, C) Box-and-whisker plot showing the fluorescence intensity of H2B::mCherry (A) and HIS-72::GFP (C) in the nucleus. n = 20. Whiskers indicate the 10th and 90th percentiles. Boxplots represent the medians and the 25th–75th percentile. *, **, and *** indicate $0.01 > p \geq 0.05$, $0.005 > p \geq 0.01$, and $p < 0.005$, respectively. (B) Bar graphs show the percent of adult animals with extra DTCs. n = 100. (D) GFP images of V5.ppp cells in *tlk-1 hira-1* mutants at the L3 stage. Arrow indicates the nucleus of V5.ppp. Images were captured by confocal microscopy at the same setting with pictures in Fig. 4A-D. Scale bars indicate 10 μm.

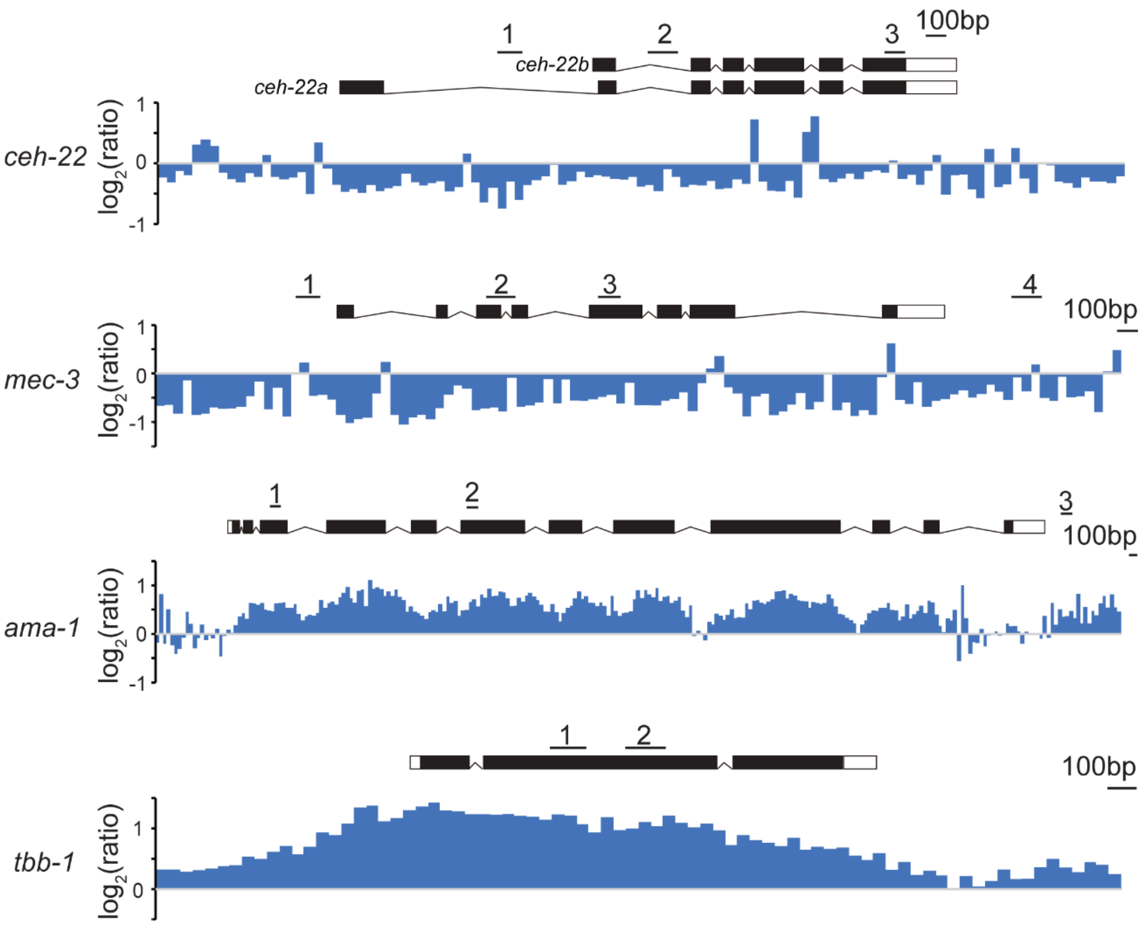

Fig. S5. ChIP-chip data in modENCODE. ChIP-chip data for HIS-72 from the modENCODE project (ID: 2536) in the region that corresponds to the *ceh-22*, *mec-3, ama-1,* and *tbb-1* loci. qPCR primers used for the DNA segments corresponding to the *ceh-22*, *mec-3*, *ama-1*, and *tbb-1* loci are shown.

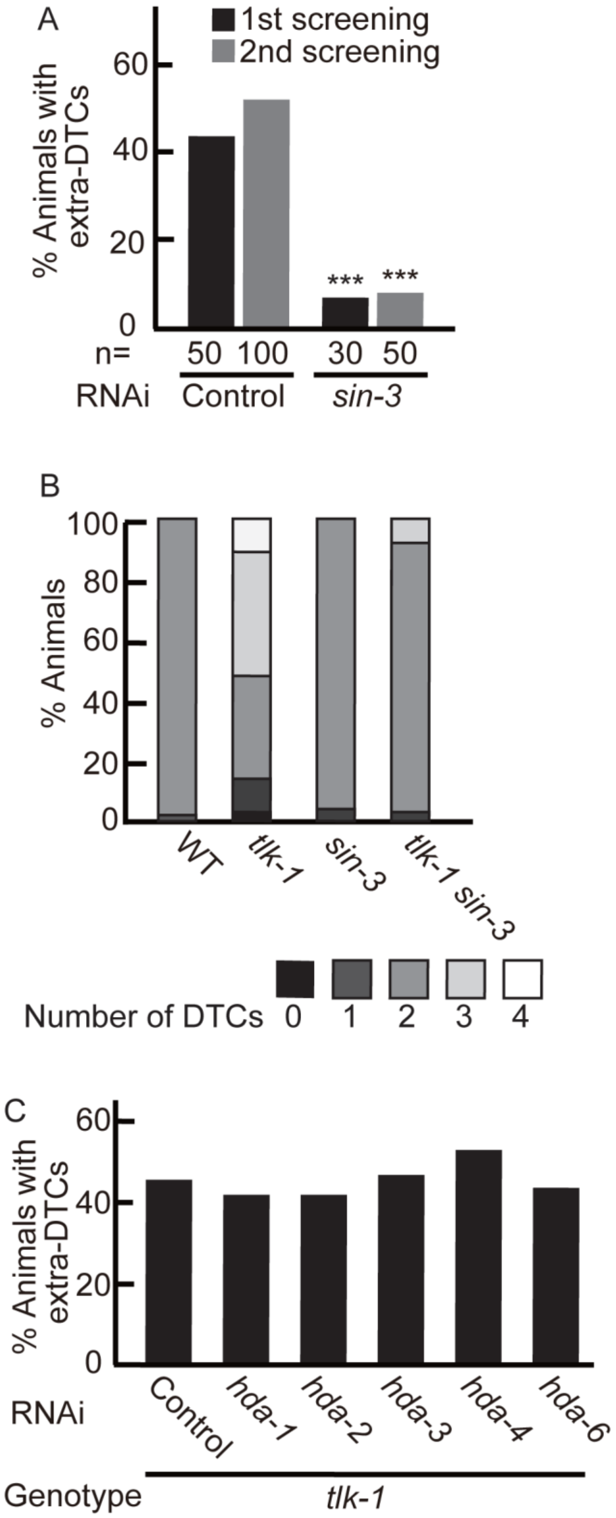
A
1st screening
2nd screening
% Animals with extra-DTCs
60
40
20
0
***
***
n=
50 100
30 50
RNAi
Control
sin-3
B
% Animals
100
80
60
40
20
0
WT
tlk-1
sin-3
tlk-1 sin-3
Number of DTCs 0 1 2 3 4
C
% Animals with extra-DTCs
60
40
20
0
RNAi
Control
hda-1
hda-2
hda-3
hda-4
hda-6
Genotype
tlk-1

Fig. S6. *sin-3* as a suppressor of *tlk-1*. (A and C) Bar graphs shows the percent of adult animals with the extra-DTC phenotype. (B) Bar graphs showing the percent of animals that had the number of DTCs indicated at the bottom. $n = 100$.

Biology Open • Supplementary information
